# Research on a Graded Self-Powered Vibration Sensor for Geological Drilling

**DOI:** 10.3390/mi16080921

**Published:** 2025-08-10

**Authors:** Jingui Zhang, Chuan Wu

**Affiliations:** 1 School of Energy and Mining Engineering, China University of Mining and Technology (Beijing), Beijing 100083, China; cjt2024031@163.com; 2 Faculty of Mechanical and Electronic Information, China University of Geosciences (Wuhan), Wuhan 430074, China

**Keywords:** triboelectric nanogenerator, self-powered, vibration sensor, geological drilling

## Abstract

Downhole vibration measurement is a key link for optimizing drilling parameters and ensuring operational safety; however, powering conventional vibration sensors reduces drilling efficiency and increases drilling costs. This paper introduces a triboelectric nanogenerator-based, graded, and self-powered vibration sensor designed for geological drilling, enabling the concurrent measurement of vibration frequency and amplitude. Experimental results show that the sensor has a measurement range for vibration frequency from 0 Hz to 11 Hz and can measure amplitude thresholds of 10 mm, 25 mm, 40 mm, and 55 mm. The measurement errors for both vibration frequency and amplitude are less than 3%, and it can operate stably under conditions of temperature below 150 °C and humidity below 90%, demonstrating good environmental adaptability. Furthermore, the sensor has self-powering capabilities, with a maximum output voltage reaching 11.8 V, a peak current of 28 nA, and a peak output power of 4 × 10^−7^ W into an external resistance of 6 × 10^7^ Ω. In contrast to conventional downhole vibration sensors, this device integrates self-powering with graded amplitude detection, enhancing its suitability for real-world drilling applications.

## 1. Introduction

Geological drilling relies on the essential technique for exploring subsurface structures and developing mineral and energy resources [1,2]. During this process, the contact of the drill bit with rock layers generates vibrations. This vibration information carries a wealth of data regarding formation lithology, drilling tool status, and drilling efficiency [3,4,5]. Therefore, the real-time and accurate monitoring of downhole vibration signals is a key link for optimizing drilling parameters and ensuring operational safety, as well as an important pathway toward achieving intelligent drilling.

Power supply schemes for downhole sensors primarily rely on downhole battery packs or wireline from the surface [6,7]. The battery-powered method is limited by battery volume and capacity. If the power is depleted during deep-well operations, the entire drill string, which can be several kilometers long, must be pulled out of the wellbore. This process is time-consuming and laborious, greatly increasing operational costs and potentially causing the loss of critical data. Although cable power can provide a continuous supply, the procurement cost of wired drill pipes is high, and their connections are highly prone to failure in the complex downhole environment. Therefore, this problem could be effectively solved if the sensors were capable of generating their power by utilizing the downhole operating conditions.

The triboelectric nanogenerator (TENG) offers a viable approach for addressing the aforementioned problem. As an emerging energy conversion and sensing technology, the core advantage of TENG stems from its capacity for efficiently transforming weak physical stimuli into electrical power, while simultaneously enabling the detection of physical signals. Consequently, it has demonstrated significant potential in the domains of energy harvesting and sensing [8,9]. In the context of power generation, TENG has been effectively employed to capture energy from human motion [10], wind [11], wave energy [12,13], and acoustic energy [14,15,16]. Since TENG generates pulse signals during its operation, and the frequency characteristics of these pulses can reflect changes in the inducing factors, it has seen extensive application in diverse sensing domains, including tactile perception [17,18], wind speed measurement [19], pressure detection [20,21,22], temperature tracking [23,24], humidity assessment [25,26], torque analysis [27], gas detection [28,29], health surveillance [30], and flow monitoring [31].

Leveraging these merits, this study puts forward a graded, self-powered vibration sensor for geological drilling constructed upon a triboelectric nanogenerator, covering the common frequency 2 Hz and vibration amplitude 30 mm in drilling operations [32,33]. The device utilizes drill string vibration to induce triboelectric power generation from its internal nanomaterials, thereby achieving self-powering. At the same time, it leverages the one-to-one correspondence between the triboelectric signal and the drill string’s vibration frequency to simultaneously measure the vibration frequency.

## 2. Structure and Working Principle

**Figure 1:**
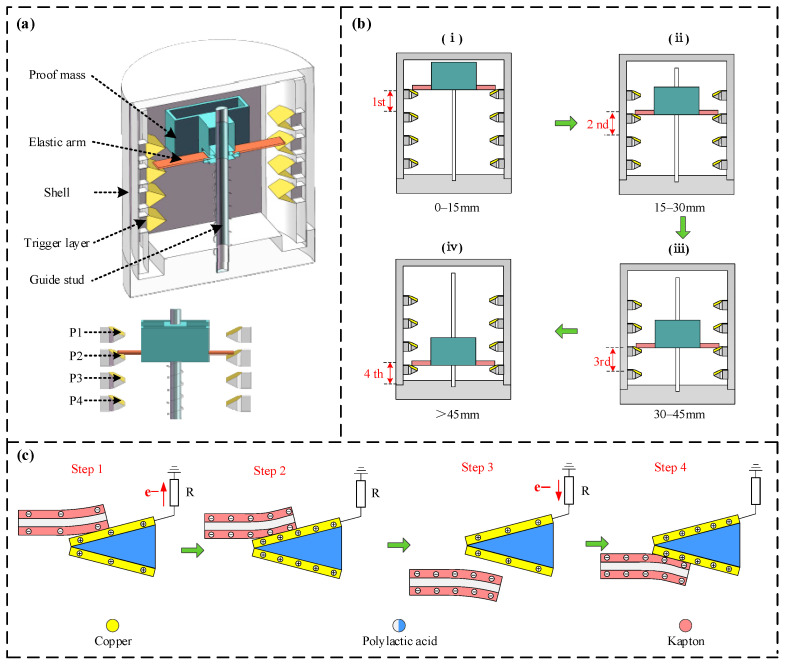
Schematic diagrams of the sensor’s structure, working principle, and operating steps. (**a**) Schematic diagram of the sensor structure; (**b**) schematic diagram of the sensor’s working principle. (i) First-level trigger schematic diagram; (ii) Second-level trigger schematic diagram; (iii) Third-level trigger schematic diagram; (iv) Fourth-level trigger schematic diagram; (**c**) schematic diagram of the principle for triboelectric signal generation.

Figure 1a illustrates the schematic design of the engineered self-powered vibration sensor. The sensor has an overall cylindrical structure with dimensions of *Φ*105 mm × *H*110 mm, and primarily consists of a fixed main body and a vibrating power-generating unit. The main body consists of four independent trigger layers arranged axially. Each trigger layer is a triangular structure with a protrusion that can be embedded into the inner wall of the housing. The layers are spaced 15 mm apart. A 30 mm × 30 mm × 0.05 mm copper (C1100, ZYTLCL Co., Ltd., Dongguan, Guangdong, China) film is attached to the surface of each trigger layer to serve as a triboelectric electrode. Each layer has a pin as a signal output terminal, with the outputs from the first to the fourth layers designated as P1, P2, P3, and P4, respectively. The vibrating power-generating unit consists of a proof mass with elastic arms made of hard cardboard and guide posts extending from its sides. The surface of the elastic arms is covered with a 60 mm × 30 mm × 0.05 mm Kapton (ZP-318, Changda Co., Ltd., Shenzhen, Guangdong, China) film, which acts as the triboelectric layer. Components such as the trigger layers, proof mass, and housing were all 3D printed using PLA (PLA001, Hubei Fangtu New Materials Co., Ltd., Jinmen, Hubei, China) material with a printing temperature of 210 °C and a 0.2 mm layer thickness.

Figure 1b illustrates the working principle of the sensor. When an external vibration occurs, the proof mass undergoes axial displacement due to the inertial force. As the displacement changes, the elastic arms sequentially make frictional contact with copper electrodes of different layers, generating triboelectric signals and thus enabling the measurement of the vibration frequency. Simultaneously, because different vibration amplitudes result in frictional contact with different electrodes (P1 to P4), four distinct amplitude thresholds can be measured by detecting which of the pins from P1 to P4 are outputting a triboelectric signal. As depicted in Figure 1b(i), for an amplitude between 0 and 15 mm, a single vibration causes the Kapton triboelectric layer on the elastic arm to make frictional contact with the copper electrode of the first layer. It then returns to its initial position under the elastic restoring force. During this process, the P1 pin outputs one triboelectric signal pulse. As shown in Figure 1b(i–iii), when the amplitude is between 15 and 30 mm, 30 and 45 mm, and greater than 45 mm, respectively, the elastic arm sequentially triggers the copper electrodes of the second, third, and fourth layers. The corresponding pins P2, P3, and P4 output their respective triboelectric signals.

The generation principle of the triboelectric signal is illustrated in Figure 1c. Since the mechanism for generating signals between the copper electrodes and the Kapton triboelectric layer is identical for all layers, the first layer serves as a representative example for this explanation. When vibration occurs, the system first transitions to the state depicted in Figure 1c—Step1. In this state, the Kapton on the surface of the elastic arm makes contact with the first-layer copper electrode. Due to the triboelectrification effect and the different electron affinities of the two materials, the Kapton becomes negatively charged, while a corresponding amount of positive charge accumulates on the copper electrode as electrons are driven to the external circuit. This generates a current flow within the circuit, and the resulting open-circuit voltage gradually increases. As the contact area expands, the system transitions to the state depicted in Figure 1c—Step2. Here, the charge transfer is finalized, the charge on both triboelectric layers is maximized, and the open-circuit voltage attains its peak. Subsequently, the system transitions to the state depicted in Figure 1c—Step3. As the elastic arm moves past the first layer, the two triboelectric layers commence separation. The electrostatic attraction for the positive charges on the copper layer diminishes, prompting a backflow of electrons from the external circuit to the copper electrode, gradually neutralizing the positive charges. This generates a reverse transfer current, which causes the open-circuit voltage to decrease. Finally, as depicted in Figure 1c—Step4, under the restoring force, the Kapton and copper layers come into full contact again, completing the charge transfer process. Throughout this cycle, current and voltage pulse signals are generated. Therefore, by using a subsequent microprocessor to read the frequency of the triboelectric signals and identifying the pin level from which the signals are generated, both the vibration frequency and amplitude can be determined.

## 3. Experiments and Analysis

**Figure 2:**
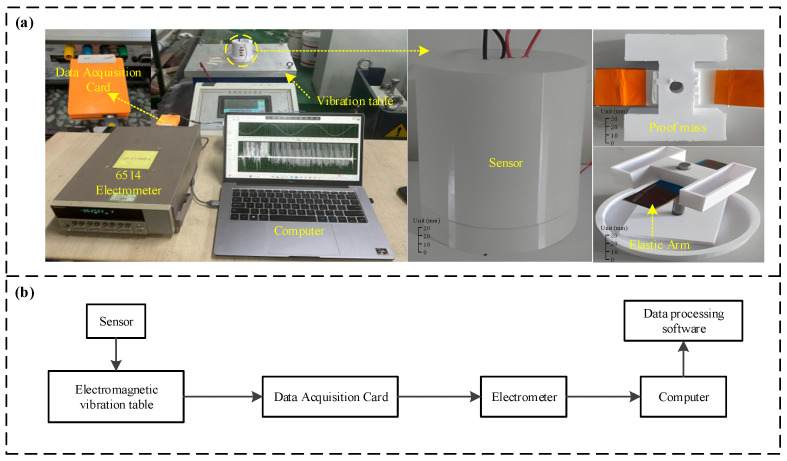
Schematic diagram of the experimental setup. (**a**) Photograph of the experimental apparatus; (**b**) schematic diagram of the experimental apparatus.

The experiment was conducted using a simulated setup as depicted in Figure 2a. The experimental apparatus consisted of a vibration controller, an electromagnetic shaker, a data acquisition card (DAQ), an electrometer, and a computer. The sensor was affixed to the electromagnetic shaker. By adjusting the parameters via the vibration controller, the amplitude and frequency of the electromagnetic shaker could be precisely controlled. As illustrated in Figure 2b, the sensor’s output data from the sensor was sequentially processed by the DAQ card and the electrometer before being relayed to the computer. This computer was equipped with software developed in LabVIEW (Version 13.0) for the real-time display and storage of sensor data. The subsequent tests were performed to assess the sensor’s sensing characteristics, measurement precision, power generation capabilities, and operational adaptability, as detailed below.

### 3.1. Sensing Performance Test Results

**Figure 3:**
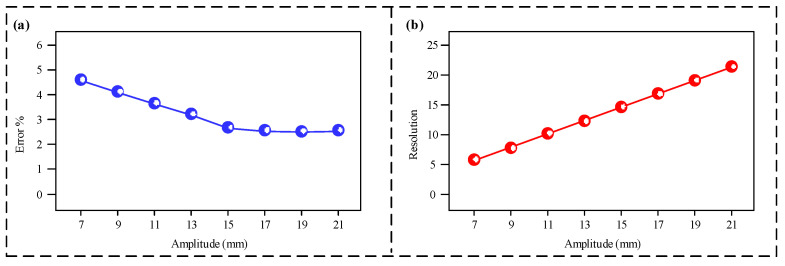
(**a**) Measurement error under different interlayer spacings; (**b**) sensor resolution under different interlayer spacings.

The effect of different interlayer spacings on the sensor’s sensing performance is shown in Figure 3. As shown in Figure 3a, with the interlayer spacing increasing from 7 mm to 15 mm, the sensor’s measurement error rate exhibits a clear downward trend, significantly decreasing from approximately 4.5% to around 2.7%. When the spacing continues to increase from 15 mm to 21 mm, the error rate remains essentially stable, showing no further significant improvement. The resolution results are shown in Figure 3b. The sensor’s measurement resolution shows a linear positive correlation with the interlayer spacing. As the spacing increases from 7 mm to 21 mm, the resolution also increases linearly from 7 mm to 21 mm. Smaller interlayer spacings enable higher measurement resolution, thus allowing for a finer differentiation of vibration amplitudes. To ensure both a low error rate and high resolution, 15 mm is selected as the interlayer spacing for all subsequent experiments.

**Figure 4:**
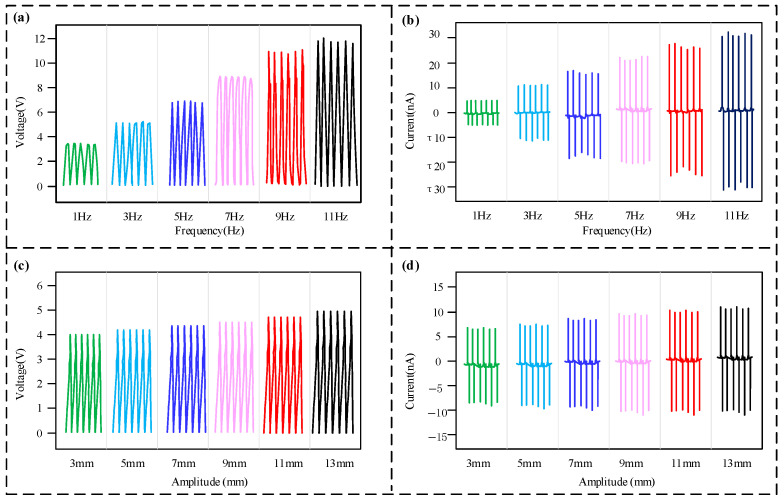
Sensing performance test results. (**a**) Voltage response to frequency variation; (**b**) current response to frequency variation; (**c**) voltage response to amplitude variation; (**d**) current response to amplitude variation.

The sensing performance test results of the sensor are shown in Figure 4, testing the sensor’s output characteristics under vibration frequencies from 1 Hz to 11 Hz and vibration amplitudes from 3 mm to 13 mm. Since the triboelectric signals generated by the different layers of the sensor were consistent, the first layer was selected as the test unit. To begin, the amplitude was fixed at 10 mm to test the performance of the sensor under different frequency conditions. Figure 4a,b illustrate that the voltage and current outputs generated by the sensor are both pulse signals, and there is a one-to-one correspondence between the vibration frequency and the number of pulses. When the frequency of vibration was raised from 1 to 11 Hz, the amplitudes of the output voltage and current both showed a distinct upward trend. When the vibration frequency reached 11 Hz, the sensor’s maximum output voltage reached around 11.8 V, while the maximum output current reached approximately 28 nA. When the vibration frequency exceeded 11 Hz, the sensor’s output waveform became irregular, and the one-to-one correspondence between the vibration frequency and the number of pulses was lost. Therefore, the measurement range for the sensor’s vibration frequency is determined to be in the range of 0 to 11 Hz. Subsequently, the performance of the sensor was evaluated at different amplitudes while the vibration frequency was fixed at 1 Hz. As shown in Figure 4c,d, with an increase in vibration amplitude from 3 mm to 13 mm, there was a corresponding steady rise in the sensor’s output voltage and current amplitudes. When the vibration amplitude was 13 mm, the sensor achieved a maximum output voltage of about 5.0 V and a maximum output current of about 11 nA, respectively. The reason for the increased signal amplitude with rising frequency or amplitude is likely that higher frequency and amplitude result in greater acceleration of the vibration shaker. This leads to a stronger impact force upon contact between the sensor’s friction layers, ensuring more complete contact and a larger frictional contact area, which in turn produces a signal with a higher amplitude.

**Figure 5:**
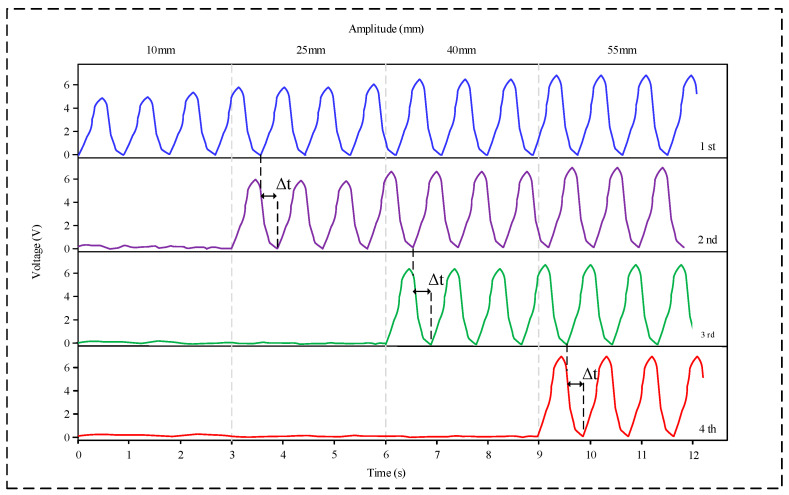
Amplitude measurement results of the sensor.

Figure 5 shows the step-wise response characteristics of the sensor when subjected to different vibration amplitudes. To ensure that the selected amplitudes can stably trigger the corresponding level, 10 mm, 25 mm, 40 mm, and 55 mm were selected in sequence. At an amplitude of 10 mm, only the first layer outputs a triboelectric signal. As the amplitude is increased to 25 mm, 40 mm, and 55 mm, the second, third, and fourth sensing layers are sequentially activated to produce stable voltage outputs, and they continue to output signals along with the previously activated layers. Furthermore, with an increase in amplitude, the magnitude of the output voltage signal also shows a progressive increase, reaching 6.1 V at an amplitude of 55 mm. These findings indicate the sensor’s capability to detect distinct amplitude thresholds at 10 mm, 25 mm, 40 mm, and 55 mm.

### 3.2. Measurement Accuracy Test Results

**Figure 6:**
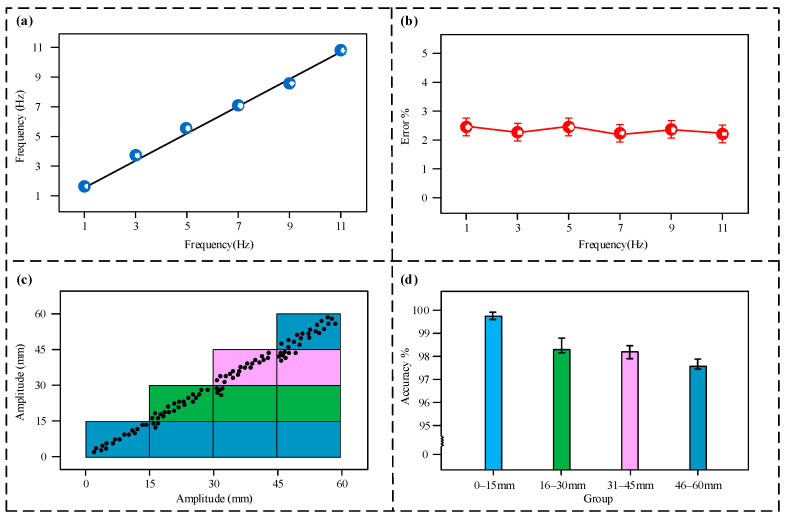
Accuracy test results. (**a**) Frequency measurement fitting curve; (**b**) measurement error at different frequencies; (**c**) error distribution map at different amplitudes; (**d**) accuracy rate at different amplitudes.

Further tests were conducted on the sensor’s precision in measurement, with the findings illustrated in Figure 6. Figure 6a illustrates that as the vibration frequency rises, the relationship between the measured frequency and the true frequency presents a linear relationship, indicating that the sensor possesses good linearity. As illustrated in Figure 6b, within the sensor’s measurement range, the frequency measurement error fluctuates within a certain range but always remains below 3%; consequently, the error in the sensor’s frequency measurement is determined to be 3%.

Figure 6c,d show the sensor’s measurement error situation at different amplitude intervals. As shown in Figure 6c, the input amplitude and the sensor’s measured values show a high degree of consistency, with the data points accurately mapped to their corresponding preset tiered intervals. As shown in Figure 6d, in the 0–15 mm interval, the sensor’s measurement accuracy rate is 99.6%. At higher amplitude intervals, the accuracy rates show a slight decrease, but all remain above 97%; therefore, the error in the sensor’s amplitude measurement also remains below 3%.

### 3.3. Power Generation Test Results

**Figure 7:**
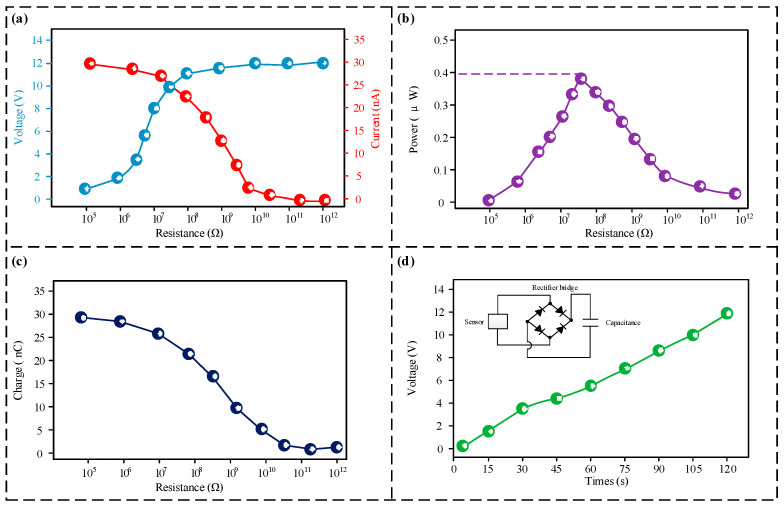
Power generation test results. (**a**) Output voltage and output current of the sensor under different loads; (**b**) output power of the sensor under different loads; (**c**) transfer charge of the sensor under different loads; (**d**) results of the sensor’s charging performance test.

The sensor also has a self-powering function during operation, so its power generation capability was further tested, with the findings displayed in Figure 7. As depicted in Figure 7a, the output voltage of the sensor gradually increases with the increasing external load resistance, while the output current decreases, which is consistent with Ohm’s law. The peak voltage and current were recorded at 11.8 V and 28 nA, respectively. Figure 7b shows the non-linear dependency of the sensor’s output power on the external load, indicating that a peak power of approximately 4 × 10^−7^ W is obtained when the external load resistance is 6 × 10^7^ Ω. Figure 7c shows the rate of transferred charge of the sensor; as the external load increases, the charge transfer rate gradually decreases, with a maximum charge of 27 nC. The experimental data for charging a 1 μF capacitor are displayed in Figure 7d. The results show that the voltage reached 11.6 V after a charging time of 120 s, indicating that the sensor has good charging characteristics.

### 3.4. Operating Condition Adaptability Test Results

**Figure 8:**
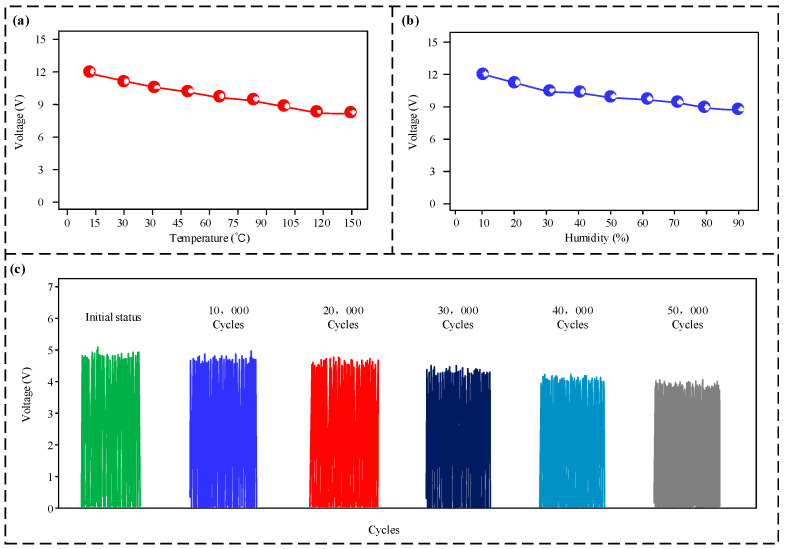
Environmental adaptability test results. (**a**) Voltage response to varying temperatures; (**b**) voltage response to varying relative humidities; (**c**) durability of the sensor’s voltage output across several operating cycles.

During drilling, as drilling progresses deeper, the temperature within the borehole rises. High temperatures can reduce the triboelectric charge transfer capability, which results in a reduction in power generation capability. To evaluate the sensor’s operating performance under different environmental conditions, experiments were performed across a temperature spectrum from 10 °C to 150 °C and a relative humidity spectrum from 10% to 90%. As demonstrated in Figure 8a,b, the experimental results indicate that the output voltage exhibits a downward trend in the output voltage as both temperature and relative humidity rise. However, throughout the entire test range, the output voltage remained above 8.6 V. The sensor’s output voltage was further tested over a varying number of operating cycles. The results, presented in Figure 8c, indicate that the output voltage’s amplitude trends downward as the number of cycles increases. However, even after 50,000 cycles, the output voltage experienced only a minor reduction, falling to about 4.6 V.

Data generated by the sensor is commonly handled by a subsequent microprocessor. Microprocessors generally utilize the Transistor–Transistor Logic (TTL) level standard to differentiate between high and low signal levels, in which a voltage input exceeding 2 V is identified as a high-level signal. While the magnitude of the sensor’s output signal decreases with rising temperature, humidity, and an increasing number of operating cycles, the reduced amplitude still substantially exceeds the TTL detection threshold. Therefore, this does not affect the later stages of signal processing. This highlights the sensor’s strong resilience to its operational surroundings and its extended operational lifespan.

## 4. Conclusions and Discussions

Utilizing the triboelectric nanogenerator principle, this paper describes the development of a hierarchical sensor for downhole vibration monitoring, capable of concurrently assessing both the frequency and amplitude of vibrations. The findings from experiments indicate that the sensor operates within a frequency detection scope from 0 to 11 Hz and can measure amplitude thresholds of 10 mm, 25 mm, 40 mm, and 55 mm, covering the common vibration frequencies and amplitudes in geological drilling. The measurement errors for both frequency and amplitude are less than 3%. Furthermore, it can operate stably under conditions of temperatures below 150 °C and humidity below 90%, demonstrating excellent environmental adaptability. The sensor also possesses self-powering capabilities, with a maximum peak voltage of 11.8 V, a peak current of 28 nA, and a peak power output of 4 × 10^−7^ W at an external load resistance of 6 × 10^7^ Ω.

The sensor offers two main advantages. First, its self-powering capability can effectively eliminate or reduce the reliance of downhole sensors on power supplies. This, in turn, reduces or eliminates the need for tripping operations to replace batteries, thereby saving on drilling costs and improving drilling efficiency. Second, the sensor’s ability to measure multiple vibration parameters allows for the concurrent assessment of both frequency and amplitude. Such data is crucial for drillers on the surface to understand actual downhole conditions, enabling them to formulate more effective drilling parameters, optimize the rate of penetration, and ensure drilling safety.

However, the sensor’s current power output is relatively low, sufficient only for its operation, and it cannot serve as an independent power source to provide real-time power for other downhole Measurement-While-Drilling systems. Future work should focus on two directions to enhance the sensor’s power output. First, by improving the sensor’s structural design, it could be able to harvest energy not only from downhole vibrations but also from the mechanical energy generated by mud flow and drill bit rotation. This would broaden the energy sources and increase the overall output capability. Second, developing new nanomaterials or improving the performance of existing ones could significantly enhance the material’s energy conversion efficiency per unit area, thereby effectively boosting the sensor’s energy output.

## Data Availability

The original contributions presented in the study are included in the article, and further inquiries can be directed to the corresponding author.
